# Cognitive styles and psi: psi researchers are more similar to skeptics than to lay believers

**DOI:** 10.3389/fpsyg.2024.1398121

**Published:** 2024-06-13

**Authors:** Marieta Pehlivanova, Marina Weiler, Bruce Greyson

**Affiliations:** Division of Perceptual Studies, Department of Psychiatry and Neurobehavioral Sciences, University of Virginia School of Medicine, Charlottesville, VA, United States

**Keywords:** paranormal belief, actively open-minded thinking, need for closure, scientific thinking, reasoning

## Abstract

**Introduction:**

Belief in psi, which includes psychic phenomena such as extra-sensory perception and post-mortem survival, is widespread yet controversial. According to one of the leading and perhaps most tested hypotheses, high belief in psi can be explained by differences in various aspects of cognition, including cognitive styles. Most of this research has been conducted with lay individuals. Here, we tested the hypothesis that academic researchers who investigate psi may exhibit different cognitive styles than lay individuals interested in psi, and are more similar to skeptics.

**Methods:**

We measured two cognitive styles—actively open-minded thinking (AOT) and the need for closure (NFC)—and assessed differences among four heterogeneous groups regarding belief in psi and involvement in related research. Specifically, our study included academic psi researchers (*N* = 44), lay individuals who believe in psi (*N* = 32), academics who are skeptics of psi (*N* = 35), and lay individuals who are skeptics (*N* = 33).

**Results:**

We found group differences in AOT (*p* = 0.003) but not in NFC scores (*p* = 0.67). *Post hoc* tests showed no significant difference in AOT scores between academics who conduct psi research (4.5 ± 0.3) and academic skeptics (4.5 ± 0.3; *p* = 0.91) or lay skeptics (4.5 ± 0.4; *p* = 0.80). The lay psi group had significantly lower AOT scores (4.2 ± 0.4) than the other three groups (*p*s: 0.005–0.04), indicating a decreased willingness to consider a range of evidence when forming an opinion, including evidence that challenges their beliefs. AOT was negatively associated with psi belief in the two skeptic groups combined (*r* = −0.29, *p* = 0.01), but not in the psi groups (*r* = −0.03, *p* = 0.78).

**Discussion:**

Our research shows that academics who work with psi differ from lay psi individuals, but not from skeptics, in actively open-minded thinking. In other words, despite their high belief in psi phenomena, psi researchers demonstrate a commitment to sound reasoning about evidence that is no different from that of skeptics.

## Introduction

*Psi* phenomena, also known as psychic phenomena, have long captivated the interest and curiosity of humanity. Psi can be defined as experiences of “information or energy transfers” that are not currently explained by known cognitive, neural, or physiological processes (Bem, 2011). Examples of psi include extra-sensory perception (ESP—the ability to perceive information without using one's physical senses) and psychokinesis (PK—the purported influence of mental processes on physical systems). Belief in psi remains relatively high among the general population, with 41% of Americans believing in ESP, 31% in telepathy, 26% in clairvoyance (the latter two both being types of ESP), 20% in reincarnation, and 73% endorsing at least one of ten purported psi phenomena (Moore, 2005). According to a more recent YouGov poll, which was representative of the US population, 63% of respondents believed they have had at least one paranormal experience (Orth, 2022).

However, despite the substantial number of individuals who hold beliefs in psi, these beliefs are often met with skepticism and dismissed as irrational and unscientific, particularly among academics (Rouder and Morey, 2011; Wagenmakers et al., 2011). A mere 4% of National Academy of Sciences members expressed favorable beliefs in ESP and PK, as revealed by a poll conducted by McConnell and Clark (1991), with academics in the physical and chemical sciences as well as psychology endorsing the most skeptical views. Similarly, McClenon (1982) reported that only 4% of American Association for the Advancement of Science members considered ESP to be an “established fact,” with 25% viewing it as a “likely possibility.” Recent data from a convenience sample of scientists, engineers, and some academics from top universities appear more favorable toward psi (Wahbeh et al., 2023). Wahbeh et al. (2023) characterized 49% of respondents as “believers” in post-mortem survival, while only 19% were “non-believers” (with the rest “uncertain”). However, the survey's framing around “consciousness surviving bodily death,” rather than as a survey of “elite scientists” (McClenon, 1982), may have skewed responses. Nevertheless, academic research on psi phenomena dates back to the nineteenth century and continues today, yielding some studies published in psychology and neuroscience journals (Bösch et al., 2006; Storm et al., 2010; Bem, 2011; Cardeña, 2018; Freedman et al., 2023). In stark contrast to academics more generally, most researchers in the field of psi appear to endorse the reality of psi, estimating its likelihood at an average of 79% (Irwin, 2014).

More recently, divergent attitudes toward psi among academics have been revealed in responses to the actual or attempted publication of psi research in scientific outlets beyond parapsychology journals. The peer-reviewed publication of a series of experiments, purportedly demonstrating modest but significant effects of the future on participants' present responses, in a high-impact psychology journal and by a highly-cited social psychologist (Bem, 2011) elicited strong reactions from many academics (Wagenmakers et al., 2011; Cardeña, 2015). These findings and their publication were variously called a “faulty result,” “an assault on science and rationality,” a failure of the peer-review process (Helfand, 2011), “crazy” and a violation of “deep belief” in science (Hofstadter, 2011), as well as a search “for the impossible” (Reber and Alcock, 2020). Based on the prevailing physicalist view of modern science, psi phenomena are deemed implausible if not impossible. This is a common criticism levied against psi research and forms the basis of its rejection as a default position of many skeptical academics (McConnell and Clark, 1991; Alcock, 2010; Reber and Alcock, 2020). Accordingly, some of these commenters, along with others, have called for censorship of such research and findings. In turn, academics engaged in psi research have described instances of academic suppression (Cardeña, 2015; Weiler et al., 2022), while calling for the open and non-dogmatic study of psi phenomena (Cardeña, 2014). Despite prevailing physicalist views, a growing number of scholars are proposing alternative non-physicalist perspectives, which could accommodate the possibility of psi phenomena (Kelly et al., 2007, 2015; Kelly and Marshall, 2021).

The controversy surrounding psi has spurred considerable research into the factors contributing to people's belief in psi. Individual differences in psi belief are associated with different factors related to demographics, personality, cognition, and culture (French, 1992; Irwin, 1993; Kennedy, 2005; Gray and Gallo, 2016; Dean et al., 2022). Compared to the other categories of predictors of, or contributing factors to belief in psi, those related to cognition stand out as particularly important. Namely, cognitive factors probe specific reasons that people may choose to interpret certain experiences as paranormal, as well as their general ability and motivation to evaluate arguments for or against the reality of psi. In addition, some cognitive factors, such as critical thinking, are more malleable compared to personality or culture. Thus, they may be more amenable to training, which could, in turn, influence psi beliefs (Wilson, 2018). As Gray and Gallo (2016) also point out, cognitive influences on psi beliefs are salient because these beliefs feature a “metacognitive component” as they “require thinking about the cognitive abilities and limitations of the human mind” (p. 242).

According to one of the leading and perhaps most tested hypotheses in this domain, high belief in psi can be explained by deficits in various aspects of cognition, including critical and scientific thinking, reasoning, and overall cognitive ability (Alcock, 1981; Irwin, 1993). This hypothesis—historically referred to as the “cognitive deficits hypothesis” of psi belief—has received support, although findings have been mixed depending on the cognitive domain, methodology, and the exact population studied (Irwin, 1993; Gray and Gallo, 2016; Dean et al., 2022). A recent study using a large battery of cognitive tasks reported that strong skeptics outperformed strong believers on measures of analytical or logical thinking, but not on memory measures (Gray and Gallo, 2016). These authors also pointed out that individual differences related to psi beliefs may indeed be viewed as differences, rather than deficits, and need not be ““good” or “bad”, nor would they necessarily imply differences in overall cognitive ability or potential for success” (Gray and Gallo, 2016). According to a recent systematic review of the decades-long literature on the association between belief in psi and cognitive functioning (Dean et al., 2022), high psi belief is most consistently associated with increased intuitive thinking (usually quick and emotion-based) and bias toward confirmatory evidence. Differences in self-reported cognitive styles—how people perceive and process information—have also been associated with different levels of psi belief (Gray and Gallo, 2016; Dean et al., 2022). In particular, greater belief in psi has been shown to correlate with lower “actively open-minded thinking”—a rational thinking disposition marked by an extensive exploration of alternatives and evidence to find the optimal answer, even if it contradicts one's beliefs (Stanovich and West, 1997; Pennycook et al., 2020; Rizeq et al., 2021). Collectively, these findings suggest that individuals may endorse psi beliefs at least partially based on emotion and insufficient consideration of conventional explanations for seemingly anomalous occurrences.

One area of inquiry that has remained unexplored is whether the associations between cognitive styles and psi belief extend to researchers engaged in academic research on psi. Based on a recent systematic review, over 60% of studies investigating the links between cognition and belief in psi have relied on undergraduate samples, and the remainder used predominantly general population samples or combined ones (Dean et al., 2022). Yet, many academic psi researchers are trained scientists and scholars (Cardeña, 2014). Even though they may exhibit a high level of endorsement of the reality of psi (Irwin, 2014), they likely differ in cognitive characteristics from the general population of lay believers. Importantly, within this group, high endorsement of psi phenomena, which would manifest as high scores on standardized measures of psi belief, may be strongly influenced by researchers' assessment of the published experimental evidence on psi (Irwin, 2014).

Cognitive styles related to evaluating evidence and reaching conclusions are of particular relevance to the controversial nature of psi, as they may contribute to how researchers (whether they are proponents or skeptics of psi) and lay individuals form beliefs about psi or engage with psi research. The literature on the “cognitive deficits hypothesis” of psi belief generally views deficient cognitive characteristics as responsible for (or at least associated with) strong psi beliefs. However, Cardeña (2011), among others, has argued that both staunch believers and skeptics who take an absolutist stance—fully endorsing or rejecting psi—have in common “intolerance for complexity and ambiguity” and unwillingness to consider other perspectives. In addition to actively open-minded thinking (AOT)—extensively investigated in relation to psi beliefs—another important albeit unexplored in this context cognitive style is the “need for cognitive closure,” often shortened as “need for closure” (NFC). NFC captures individual differences in the motivation to seek closure during information processing when faced with a decision or judgment (Webster and Kruglanski, 1994). Specifically, NFC measures the tendency to quickly settle on an answer, even if it is not correct or optimal, to end further information processing, indicating a preference for any answer, as compared with confusion and ambiguity (Webster and Kruglanski, 1994; Neuberg et al., 1997). Individuals who score high on measures of NFC tend to be more “closed-minded, resistant to information inconsistent with their firm opinions, and reluctant to have their knowledge challenged” (Roets et al., 2015).

In this study, we investigated differences in cognitive styles (AOT and NFC) among four heterogeneous groups regarding belief in psi and attitudes toward and involvement in related research: academic psi researchers, lay psi believers, academic skeptics, and lay skeptics. This research sought to shed light on two main questions: (1) Are psi researchers different from lay believers in how they approach knowledge, evidence, and ambiguity? (2) Are psi researchers—who engage in this research as a legitimate scientific pursuit which can yield observations incompatible with physicalist views—different than skeptics with similar academic and scholarly training in terms of considering inconsistent evidence and their motivation to search for the “correct” answer? We assessed AOT, NFC, as well as psi beliefs and psi experiences via self-report questionnaires, and examined differences between groups. We hypothesized that psi researchers would demonstrate high psi belief akin to lay believers, yet cognitive styles more similar to those of academic skeptics than lay believers. This is because psi researchers (a) typically are academics trained in the principles of scientific inquiry and rigor, including critical evaluation of hypotheses; and (b) they likely developed their views on psi through a different process—e.g., evaluating the outcomes of research, including their own—than lay believers.

## Materials and methods

### Participants and recruitment

The study included four participant groups: 44 individuals who have engaged in academic psi research (academic psi group); 32 individuals who identify as psi believers or enthusiasts but are not engaged in academic psi research (lay psi group); 35 individuals who are academic or professional skeptics of psi (academic skeptic group); and 33 individuals who are skeptics of psi but not academics (lay skeptic group).

The academic psi group was recruited from mailing lists dedicated to parapsychology (e.g., “Survival Net,” an invitation-only international electronic mailing list for discussion of survival of consciousness, non-local consciousness, and related topics) and institutions focusing on related research (e.g., the Institute of Noetic Sciences and the Windbridge Research Center). In addition, we emailed the study invitation to psi researchers who may not be members of these lists or organizations. Fifty-three individuals consented to and finished the questionnaire within the academic psi group. The final analysis sample consisted of 44 academic psi researchers, excluding 7 respondents who have not conducted psi research and two repeat responses. Among this group, 81.8% identified as Caucasian; 6.8% as Asian; 6.8% as Hispanic; and 11.5% as other (participants could make multiple selections). Additional demographic characteristics for this and other groups are provided in Table 1.

**Table 1:** Demographic characteristics, psi beliefs and experiences, and cognitive styles by participant group.

		**Academic psi *N =* 44**	**Lay psi *N =* 32**	**Academic skeptics *N =* 35**	**Lay skeptics *N =* 33**	**Test statistics**	***P*-values**
		**Mean/Median** ±**SD, or %**	**Mean/Median** ±**SD, or %**	**Mean/Median** ±**SD, or %**	**Mean/Median** ±**SD, or %**		
Demographics	Sex					$\chi_{\left(3\right)}^{2}$ = 7.9	0.048^*^
	Woman	22.7%	50%	25.7%	42.4%		
	Man	72.7%	50%	74.3%	54.5%		
	Decline to answer	4.5%	0%	0%	3.0%		
	Age^∧^	60.0/59 ± 14.4	51.3/50 ± 12.7	65.5/66 ± 11.8	53.7/58 ± 13.1	*F*_(3, 138)_ = 8.1	< 0.0001
	Education^#^					$\chi_{\left(9\right)}^{2}$ = 109.2	< 0.0001
	High school/less than college	0%	43.8%	2.9%	24.2%		
	College/some graduate studies	4.6%	28.1%	8.6%	51.5%		
	Master's degree	7.0%	25.0%	8.6%	24.2%		
	Doctoral degree	88.4%	3.1%	80.0%	0.0%		
NEBS	Beliefs	77.0/80 ± 18.7	89.1/91 ± 9.7	8.8/6.6 ± 6.9	9.6/7 ± 10.8	*F*_(3, 140)_ = 387.4	< 0.0001
	Experiences	47.1/45 ± 22.1	61.7/66 ± 28.5	7.4/6.2 ± 6.8	8.1/7 ± 9.8	*F*_(3, 140)_ = 72.0	< 0.0001
Cognitive styles	AOT^∧^	4.5/4.5 ± 0.3	4.2/4.2 ± 0.4	4.5/4.5 ± 0.3	4.5/4.6 ± 0.4	*F*_(3, 138)_ = 4.8	0.003
	NFC	3.0/3.1 ± 0.8	3.2/3.4 ± 0.7	3.1/3.1 ± 0.7	3.2/3.1 ± 0.7	*F*_(3, 140)_ = 0.5	0.67

SD, Standard deviation; ^*^Statistical test after excluding “decline to answer” observations; ^∧^N = 42 for the academic psi group; ^#^N = 43 for the academic psi group; NEBS, Noetic Experiences and Beliefs Scale.

The lay psi group was recruited from large Facebook groups of interest in paranormal topics and through organizations with a focus on psi phenomena and/or psi research (e.g., the Monroe Institute). All 32 respondents in this group who consented to the study finished the questionnaire. Among lay believers, 90.6% identified as Caucasian; 6.2% as Asian; 3.1% as Hispanic; and 3.1% as Other.

Academic skeptics were recruited by personalized email invitation to Fellows of the Committee for Skeptical Inquiry (CSI), who are elected to this position by the organization's Executive Council “for their distinguished contributions to science and skepticism” (Skeptical Inquirer, 2021). Specifically, election requires “outstanding contributions” to (1) a scientific discipline (2) the “communication of science and/or critical thinking,” and (3) to the skeptical movement (Skeptical Inquirer, 2021). In addition, we invited by email some academics who were not part of this list but who have been active contributors against psi research. All 35 respondents in this group who consented to the study finished the questionnaire. Among academic skeptics, 94.3% identified as Caucasian; 2.9% as Hispanic; and 5.7% as other.

Participants in the lay skeptic group were recruited via email invitations to some individuals who have contributed to the Skeptical Inquirer blog (https://skepticalinquirer.org/)—a magazine published by the CSI—who also forwarded the study invite to fellow skeptics. In addition, we recruited participants through a Facebook group focused on skepticism.^1^ In the middle of recruitment efforts, we also posted a call for participants on the Skeptical Inquirer blog with the assistance of the magazine. Interested individuals were able to sign up for the study, after endorsing inclusion criteria, and were informed that some will be selected at random to participate. Subsequently, we discovered that most of the randomly selected individuals who endorsed being skeptics provided answers about psi beliefs that resembled those of believers, possibly influenced by the promised gift card. The final sample consisted of 33 lay skeptics, after excluding one respondent who consented to but did not finish the questionnaire and 5 “fake” skeptics. Although we do not report these analyses here for brevity, treating these “fake” skeptics as lay believers did not substantially change the results reported in this article. Among lay skeptics, 87.9% identified as Caucasian; 12.1% as Hispanic; and 3.0% as Asian.

Inclusion criteria common to all groups included being at least 18 years of age and fluent in English. In both skeptic groups, participants were explicitly asked to endorse being “a skeptic of the paranormal and fringe science.” Participants in the lay psi group were asked to endorse being a “believer in the paranormal or psi enthusiast.” Potential participants in the academic psi group were asked whether they are psi or parapsychology researchers who are producing or have produced empirical or theoretical psi research that would be publishable in an academic journal. Participants in all groups were convenience samples from the respective populations, with a desired minimum sample of 30 in each group. The sample size was chosen based on population limitations, particularly in the two academic groups, and to achieve sufficient numbers for the central limit theorem to relax distributional assumptions.

It is important to clarify conceptual distinctions in psi research engagement between academic psi researchers and academic skeptics. Academic psi researchers typically view psi research as a legitimate scientific pursuit, conducting research to document and understand purported psi phenomena. In contrast, academic skeptics who are fellows of the CSI promote scientific skepticism, which generally takes the position that psi phenomena do not exist and considers investigations into such phenomena to be “pseudoscience.” Aligned with scientific skepticism, one could engage in psi-related research to disprove or debunk psi phenomena or the merit of such scientific pursuits or specifically to investigate beliefs in psi as irrational and people who hold such beliefs as cognitively deficient.^2^

### Procedure

Each group of participants completed a single online questionnaire administered via Qualtrics (Provo, Utah, USA), a secure survey platform with a site license provided by the University of Virginia. The study protocol was approved by the University of Virginia's Institutional Review Board for Social and Behavioral Sciences (protocol #3926). Participants provided consent electronically at the beginning of the survey. Each participant was offered a 10 USD Amazon gift card for completing the survey and asked to provide an email if they were interested in receiving the compensation.

### Online questionnaire

The online questionnaire consisted of 62 items, not including the consent question, control questions about eligibility, and the gift card question. Six of these questions were shown conditionally based on answers about education and involvement in academic research (psi or other). Forty-five of the questions pertained to the three self-report measures described in the next subsection. We inquired about participants' socio-demographic characteristics, including standard questions about age, gender, race, country of residence, education, employment status, and religious preference or affiliation. In addition, the questionnaire included items about participants' professional involvement in psi research, including length of involvement, affiliations, and number of published scholarly articles. Respondents were given the opportunity to provide open-ended comments at the end of the survey. The questionnaire allowed completion in multiple sittings and going back to previous items.

### Measures

#### Noetic experiences and beliefs scale

The Noetic Experiences and Beliefs Scale (NEBS) is a novel 20-item self-report questionnaire assessing psi beliefs and psi experiences as separate constructs (Wahbeh et al., 2020). The questionnaire consists of questions about 10 anomalous or extraordinary domains, each evaluated for the respective degree of belief and experience of the participant. For each domain, questions are asked as follows: “I believe that my consciousness is not limited by my physical brain or body” (an example of a belief question) and “I have personally had this experience” (for experience). Responses are reported on a visual analog scale ranging from “disagree strongly” to “agree strongly” for beliefs, and “never” to “always” for experiences, with numerical equivalents between 0 and 100. In the original validation study, the NEBS demonstrated excellent internal consistency (Cronbach's α: 0.90 and 0.93 for belief and experience subscales, respectively), good test-retest reliability at 1 month (*r* = 0.83 and *r* = 0.77 for belief and experience subscales, respectively) and the latent two-factor structure of beliefs and experiences was supported via confirmatory factor analysis (Wahbeh et al., 2020). Cronbach's α in this sample was 0.98 for the belief and 0.96 for the experience subscales, which may suggest possible redundancy of some of the survey items. When examined separately, the skeptic groups show lower α values (0.78 for beliefs and 0.76 for experiences) than the psi groups (0.92 for beliefs and 0.93 for experiences).

#### Actively open-minded thinking

To assess actively open-minded thinking as a dispositional cognitive trait, we used a 10-item self-report AOT scale, suggested as the most valid and reliable version by the Society for Judgment and Decision Making at the time of study design in November 2020 [http://sjdm.org/; Baron et al. (2015) used an 8-item version; Baron (2019) used an 11-item version]. A composite scale measuring AOT was originally developed by Stanovich and West (1997), based on a conceptualization of the trait by Baron (1985). In the following decades, the measurement of AOT has undergone significant changes, as outlined by Stanovich and Toplak (2023), including adding items tapping into additional facets of AOT, shortening the scale, and refining questions to minimize bias. The version of the scale used here includes items such as “Willingness to be convinced by opposing arguments is a sign of good character” and “Changing your mind is a sign of weakness” (reverse-scored), rated on a five-point scale ranging from “1 = completely disagree” to “5 = completely agree” and including a “3 = neutral” option. Higher scores on this scale indicate greater actively open-minded thinking. The AOT scale demonstrated adequate internal consistency in this sample with a Cronbach's α of 0.73.

#### Brief need for closure scale

To assess the need for closure as a dispositional trait, we used a brief 15-item Need for Closure Scale (Roets and Van Hiel, 2011). This self-report scale was developed and validated as an abridged version of a modified NFC scale (Roets and Van Hiel, 2007). Even though the revised scale incorporated all five original facets of Order, Predictability, Ambiguity, Closed-mindedness, and Decisiveness, it was validated via principal component analysis as a one-dimensional measure tapping a unitary construct (Roets and Van Hiel, 2007). The brief NFC scale showed good internal consistency (Cronbach's α = 0.87), adequate test-retest reliability at 1 month (*r* = 0.79), and good convergent and divergent validity, showing psychometric properties that were similar to those of the revised full scale (Roets and Van Hiel, 2011). The brief NFC includes items such as “I dislike questions which could be answered in many different ways” and “I do not usually consult many different opinions before forming my own view,” rated on a six-point scale ranging from “1 = strongly disagree” to “6 = strongly agree,” without a neutral option. Higher scores on this scale indicate a greater need for closure. Internal consistency in this sample was also good, with a Cronbach's α of 0.83.

### Statistical analysis

Descriptive statistics were presented as means, medians, and standard deviations for continuous variables and percentages within groups for categorical variables. ANOVA was used to test for group differences in psi beliefs/experiences, cognitive styles, and participants' age, without adjusting for covariates. Analysis of covariance (ANCOVA) was used to test for these group differences while including covariates as additional independent variables in the models. Specifically, given previous findings of age and education associations with AOT and NFC (Kossowska et al., 2012; Chen, 2015; Edgcumbe, 2022), and differences in these demographic variables between groups in this study, we assessed group differences while adjusting for age and education as an ordinal variable.

Pairwise differences after a significant ANOVA/ANCOVA group effect were assessed via Tukey-adjusted *post hoc* tests. Effect sizes for the main effects of ANOVA/ANCOVA were presented as eta squared and partial eta squared. Pearson's chi-squared tests were used to test the association between group and categorical variables like sex and education. Pearson's correlation coefficients were used for all bivariate correlation analyses. All data management and statistical analyses were conducted using SAS 9.4 (SAS Institute, Cary, NC).

### Power analysis

Given the sample size limitations in this study, we conducted a *post hoc* sensitivity power analysis using G^*^Power Version 3.1.9.7 (Faul et al., 2007). A one-way between-subjects analysis of variance with 144 participants and four groups would be sensitive to effects of η^2^ = 0.07 or *f* = 0.28 (conventionally, a medium effect size), assuming 80% power and an alpha of 0.05. In other words, the study would not be able to reliably detect effects smaller than η^2^ = 0.07. Note that G^*^Power outputs effect sizes in Cohen's *f* , which has been converted to η^2^ according to Cohen (2009).

To our knowledge, there are currently no established benchmarks in the particular groups included in this study for effect sizes or expected mean levels for the cognitive styles under examination. However, some prior research may inform reasonable estimates of group differences in AOT that are associated with objective measures of argument evaluation. Stanovich and West (1997) administered an argument evaluation test and various cognitive style measures to a large group of participants. The authors developed an index of one's ability to evaluate the quality of an argument independently of one's prior beliefs about an issue. Classifying participants into groups based on their high or low reliance on argument quality when evaluating a proposition, Stanovich and West (1997) reported that the high reliance group showed significantly higher disposition toward AOT compared to the low reliance group. Using descriptive statistics from the article, we calculated that the effect size of this difference approximates a Cohen's *d* of 0.51 (equivalent to *f* of 0.25 or η^2^ of 0.06). For the NFC scale, we could not identify studies directly addressing associations with relevant objective measures. However, associations between NFC and measures relevant to evidence processing, such as intolerance for ambiguity, need for cognition, and dogmatism, fall in the range of 0.58–0.60 in terms of Cohen's *d* (*f* : 0.29–0.30 or η^2^ around 0.08) (Webster and Kruglanski, 1994). The magnitude of such AOT and NFC effects are in line with what our sample allows us to detect.

## Results

### Demographic characteristics

Due to the nature of the participant groups, differences in demographics were expected. In terms of education, the two groups consisting primarily of academics—the academic psi and academic skeptic groups—had achieved higher education, on average, than the lay believers and skeptics (Table 1). In addition, the academic groups differed from the lay groups in terms of sex and age. Notably, the sex ratio among the academic psi group exactly mirrors previously published estimates (Mayer et al., 2022). Academic skeptics were the oldest, on average, and differed significantly from both the lay psi group (*p* = 0.0001) and the lay skeptic group (*p* = 0.002), but not from the academic psi group (*p* = 0.27). Participants in the academic psi group were significantly older than those in the lay psi group (*p* = 0.03) but not the lay skeptic group (*p* = 0.17).

### Group differences in psi beliefs and experiences

**Figure 1:**
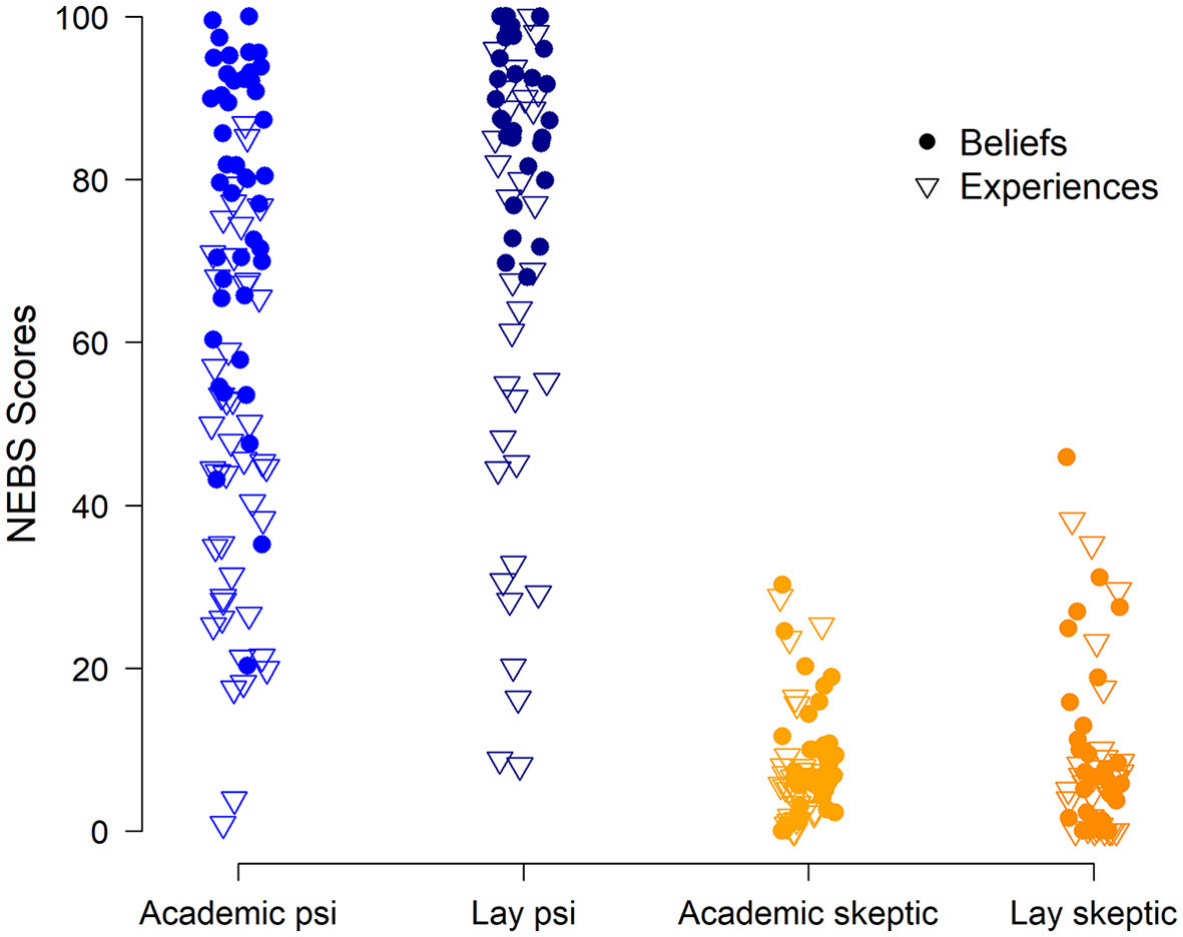
Dot plot showing Noetic Experiences and Beliefs Scale (NEBS) scores (Y-axis) by study group (X-axis). Academic psi (*N* = 44) and lay psi (*N* = 32) groups showed higher belief and experience scores than the academic skeptic (*N* = 35) and lay skeptic (*N* = 33) groups.

As anticipated, there were differences between the groups on both psi beliefs (*p* < 0.0001, η^2^ = 0.89) and experiences (*p* < 0.0001, η^2^ = 0.61; Table 1, Figure 1), as measured by the NEBS. *Post hoc* tests revealed that the academic psi and lay psi groups have significantly higher psi belief scores than both skeptic groups (*p*s < 0.0001 for all four comparisons). Psi belief scores did not differ significantly between the two skeptic groups (*p* = 0.99). While both psi groups showed high levels of belief, participants in the academic psi group had significantly lower belief scores, on average, than those in the lay psi group (*p* = 0.0005), and showed higher variability in their beliefs.

The pattern of group differences in experience scores was identical to that for belief scores. The academic psi and lay psi groups had significantly higher psi experience scores than both skeptic groups (*p*s < 0.0001), but differed from each other, with the academic group reporting lower levels of psi experiences than the lay group (*p* = 0.007). Psi experience scores did not differ significantly between the academic and the lay skeptic group (*p* = 0.99).

Scores on the psi beliefs and psi experiences subscales were significantly correlated with each other in all four groups, with the weakest correlation occurring in the academic psi group (*r* = 0.48, *p* = 0.001) and the strongest in the lay skeptic group (*r* = 0.78, *p* < 0.0001).

### Group differences in cognitive styles

ANOVA revealed group differences in AOT scores (*p* = 0.003, η^2^ = 0.09), but not in NFC (*p* = 0.67, η^2^ = 0.01). *Post hoc* tests showed no significant difference in AOT between the academic psi and academic skeptic groups, which lines up with our original hypothesis (*p* = 0.91). The academic psi group was also not significantly different in AOT scores from lay skeptics (*p* = 0.80). The lay psi group had significantly lower AOT scores than the academic psi (*p* = 0.04), academic skeptic (*p* = 0.01), and lay skeptic (*p* = 0.005) groups.

ANCOVA adjusting for age and education revealed group differences in AOT [*F*_(3, 135)_ = 3.68, *p* = 0.01, η^2^ = 0.08], but not in NFC [*F*_(3, 136)_ = 0.58, *p* = 0.63, η^2^ = 0.01]. Similarly to unadjusted analyses, *post hoc* tests showed no significant difference in AOT between the academic psi and the academic skeptic (*p* = 0.98) or lay skeptic (*p* = 0.26) groups. The lay psi group had significantly lower AOT scores than the lay skeptic group (*p* = 0.009) but was no longer significantly different from the academic psi (*p* = 0.94) and academic skeptic (*p* =0.82) groups.

### Correlations between psi beliefs and cognitive styles

**Figure 2:**
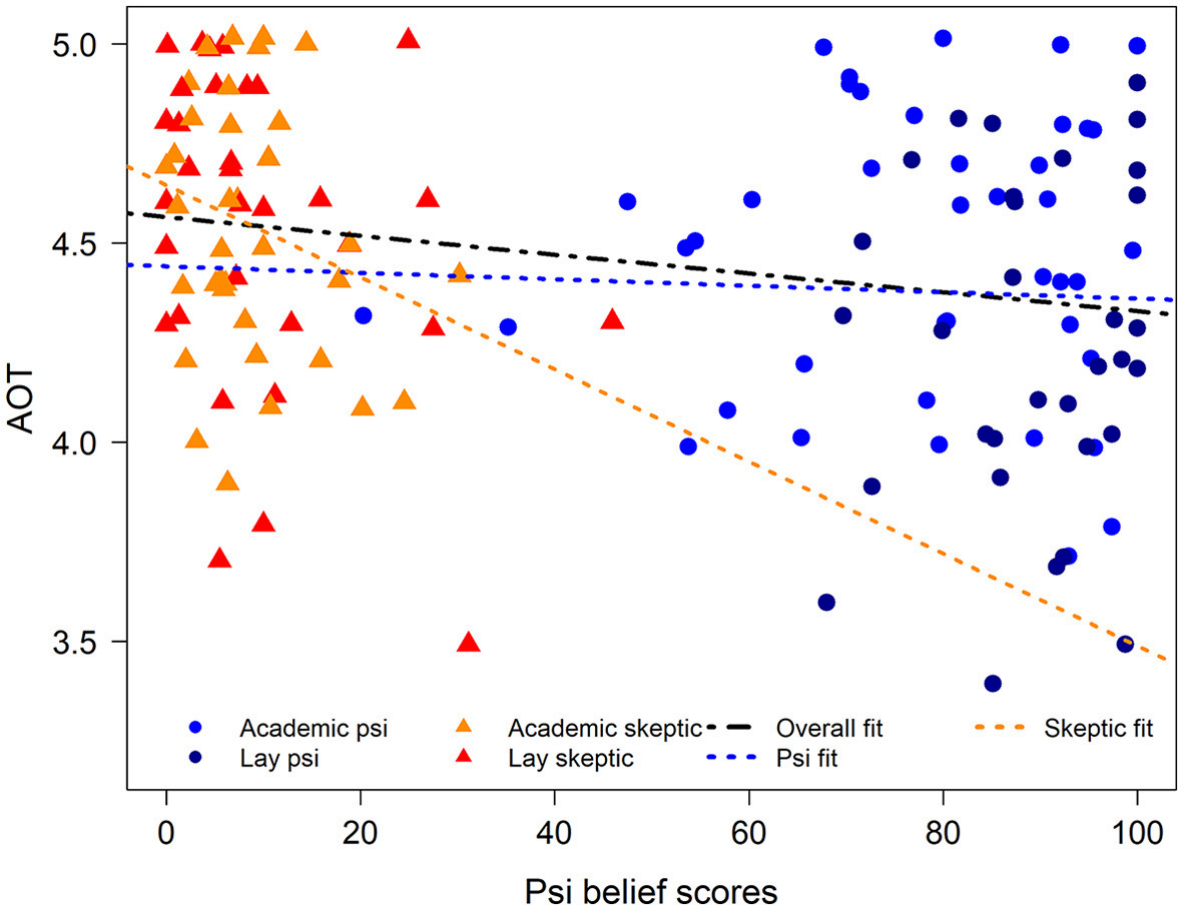
Scatterplot for the relationship between psi belief scores and actively open-minded thinking (AOT). Scores for each of the four groups are shown in different colors. Lines of “best fit” for the relationship are shown separately for the total sample (overall fit), the psi groups combined (psi fit), and the skeptic groups combined (skeptic fit). A small amount of jitter was added to values on both axes to facilitate visualization of overlapping points. AOT is negatively correlated with psi belief in the total sample and among the skeptic groups, but not the psi groups.

Next, we examined the relationship between cognitive styles and belief in and experience with psi across the entire sample. Belief and experience scores were significantly negatively correlated with AOT (*r* = −0.24, *p* = 0.004; *r* = −0.22, *p* = 0.01, respectively; Figure 2 for belief scores), such that higher NEBS scores are associated with lower endorsement of AOT principles. However, belief and experience scores were not correlated with NFC (*r* = −0.04, *p* = 0.62; *r* = −0.14, *p* = 0.10). When examining the effect separately for psi vs. skeptic groups, it appears that the significant associations between AOT and psi belief scores are driven by the skeptics, at the lower range of belief and experience scores. Specifically, AOT and psi beliefs and experiences were significantly correlated in the two skeptic groups combined (*r* = −0.29, *p* = 0.01; *r* = −0.27, *p* = 0.02, respectively), but not in the two psi groups (*r* = −0.03, *p* = 0.78; *r* = −0.04, *p* = 0.75).

### Narrative data

Although not necessarily representative, certain comments by participants help contextualize differences and similarities between the groups. Some researchers in the academic psi group commented on the appropriateness of asking about *belief* in psi presumably as the basis of one's interest in purported psi phenomena. For example, one PhD-level psychologist involved in the research for 5–10 years wrote: “*For me, it is not about my ‘beliefs' it is about the evidence.”* Another respondent wrote: “*This survey was oddly worded if the target audience was research scientists. I don't ‘believe' things. I take a flexible position that is constantly reevaluated based on the available data.”*

Despite their differences in assessments of psi compared to psi researchers, some academic and lay skeptics stated an openness to the possibility of psi if the right evidence or explanation is presented. One neuroscientist wrote: “*I am open to the idea that there are aspects of the physical world that we don't understand […], but once those were explicated they would then be understood, modeled, reproducible and would fall into the category of physical world. Thus my statement that ‘I have no belief in the non-physical'.”* The idea of openness and the necessity of evidence of psi being reproducible was echoed by others, exemplified by this comment from a lay skeptic: “*As a skeptic, I need to have an open mind to all possible answers. […] I am open to new evidence but it needs to be valid and reproducible evidence.”* Notably, several skeptics suggested that personal experience cannot be construed as evidence: “*I would need some pretty indisputable evidence, even if I thought something may have happened to myself.”*

Compared to the other three groups, participants in the lay psi group were most likely to mention specific psi experiences they may have had—sometimes detailing their different types and duration—as well as how those experiences directly influenced their beliefs in psi. Some participants in this group specifically commented on the role of logic and evidence in their perceptions: “*Myself, I'm very logical and what I experience of the energetic and spiritual world to me does not defy the science or contradict logic. If I can't understand the spiritual and energy logically, I wouldn't be involved in it.”* Another wrote: “*Paranormal Investigation is all about making sure there's concrete evidence.”* Additionally, several respondents in this group commented on how they and people in general can develop the ability to experience psi phenomena.

## Discussion

In this manuscript, we aimed to test the hypothesis that academic psi researchers may exhibit different cognitive styles compared to lay individuals interested in psi, but similar to skeptics. We compared two cognitive styles relevant to evidence processing and judgments—actively open-minded thinking and the need for closure—between heterogeneous groups in terms of belief in psi and attitudes toward and involvement in psi research. Specifically, we included two groups of academics—psi researchers and skeptics—as well as two lay groups of participants who either believe in psi or are skeptics of it.

### Comparing the academic psi and academic skeptic groups

A primary focus of this investigation was to compare academics and researchers who are engaged in studying psi and those who take a skeptical position toward this field and its underlying phenomena. Not surprisingly given their different engagement with psi, researchers in the field reported significantly greater belief in and perceived experience with psi phenomena compared to academic skeptics, echoing prior findings (Blackmore, 1989; Irwin, 2014). However, as hypothesized, psi researchers and academic skeptics showed no difference in the cognitive styles of AOT and NFC. Together, these findings suggest that these two groups that are philosophically and empirically at odds with each other regarding evidence for psi phenomena nonetheless do not differ in their endorsement of the principles of “good” thinking about evidence (Baron et al., 2015). These encompass actively seeking out evidence that contradicts one's beliefs, being willing to update one's beliefs in light of new evidence, and being comfortable with ambiguity (Stanovich and Toplak, 2023). Additionally, the two groups did not differ in the extent to which they form opinions quickly to avoid ambiguity (Roets and Van Hiel, 2011).

Supporting the notion that these two groups are not entirely dissimilar, a previous survey comparing the views of psi researchers and skeptics revealed several areas of agreement (Blackmore, 1989). Among those were the acknowledgment of contributions of psi research to other fields (including psychology, statistics, and philosophy of science), potential concerns about lack of replicability in the field, and general “open-mindedness and doubt” when evaluating evidence (Blackmore, 1989). On the other hand, Blackmore (1989) highlighted an important difference between the two groups in their interpretation of research that aims to establish the reality of psi. Namely, skeptics indicated that they considered only laboratory experiments relevant as evidence of psi, and even then, they found them unconvincing. In contrast, psi researchers indicated that they found the totality of psi research—including experiments and spontaneous cases (e.g., near-death experiences)—to be relevant and convincing. A more recent survey with members of the Parapsychological Association (PA) substantiated this, revealing that overall they deemed the cumulative experimental psi evidence most persuasive (79% combined for “strongly” or “extremely” persuasive) (Irwin, 2014). However, PA members also viewed spontaneous cases as well as personal experience as persuasive, though to a lesser extent (Irwin, 2014). This divergence was also reflected in our narrative data, where skeptics (both academic and lay) singled out the importance of reproducible, experimental evidence for psi, which they consider to be lacking, and discounted the relevance of personal experience.

Despite historic disagreement and even vitriol, members of the two groups have previously conducted successful and informative “skeptic-proponent collaborations” (Hyman and Honorton, 1986; Schlitz et al., 2006), highlighting areas of agreement including methodological improvements for future psi studies. These collaborative efforts have been acknowledged as valuable to the field by the psi researcher community (Roe, 2017; Parapsychological Association, 2023). Over time, such engagements have contributed to a shift in the nature of the disagreement, moving from disputes about “the existence of [anomalous] effects to their interpretation” (Hyman and Honorton, 1986; Honorton, 1993).

Ultimately, our goal here is not to debate the merits of psi research and evidence. Their interpretation and value have generated significant and long-lasting debates between psi researchers and skeptics (Krippner and Friedman, 2010). The data we present suggest that, despite these differences and the perception of psi researchers as “poor thinkers” and of skeptics as uninformed dogmatists (Roe, 2017), psi researchers and skeptics may not differ considerably in their thinking styles, as is commonly expected.

In the context of these potential similarities, it is interesting to consider what drives psi researchers to engage in this research, even though our study was not designed to directly answer this question. We observed that academic psi researchers endorsed significantly higher psi beliefs, as well as psi experiences, compared to academic skeptics. These experiences attributed to psi processes can serve as a possible motivator of research interests in psi, as 53% of members of the PA found personal experience “strongly” or “extremely” persuasive as a source of evidence for the reality of psi (Irwin, 2014). Indeed, scientists' own extraordinary and spiritual experiences have in some cases prompted significant career changes, including shifting one's work toward exploring the nature of consciousness (Woollacott and Shumway-Cook, 2023).

Speculatively, it is conceivable that factors beyond cognitive ones, such as personality, may influence researchers' inclination to investigate psi phenomena. One possible contributing factor is openness to experience, which is positively correlated with both psi beliefs (Chauvin and Mullet, 2021) and psi experiences (Zingrone et al., 1998-1999). This dimension of personality can be accompanied by unconventional attitudes and interest in novel ideas (McCrae, 1993). Among scientists, openness to experience is specifically associated with conducting “boundary-spanning” and perhaps riskier research (Bateman and Hess, 2015), which undoubtedly applies to psi research.

### Comparing the academic psi and lay psi groups

Another important and purposeful comparison in this study was to assess cognitive style differences between academic psi and non-academic lay psi individuals. Although most psi researchers would identify as “believers” (Blackmore, 1989; Irwin, 2014), these groups are fundamentally distinct in terms of their academic interest in purported psi phenomena, their familiarity and involvement with psi research, and their ability to engage with it. As anticipated, both groups showed high levels of belief in and experience with psi compared to skeptics, with the lay psi group nonetheless scoring significantly higher than the academic psi group. In contrast, the academic psi group showed greater levels of AOT compared to the lay group, indicating a greater willingness to consider a range of evidence when forming opinions, including evidence that contradicts their beliefs. This difference did not hold after accounting for educational and age differences between the groups. Nonetheless, we contend that these differences, especially in education, are defining features of the two groups. As such, they are relevant and should not be fully eliminated, for a fair comparison of differences between academic psi researchers and lay psi believers. Notably, after accounting for age and education, the lay psi group also did not differ from the academic skeptic group in terms of AOT.

Despite both groups exhibiting high belief in and perceived experience with psi, they may ultimately differ in how these beliefs originated or strengthened. Beliefs and experiences were positively correlated in both psi groups, but this correlation was stronger in the lay psi group. Notably, many lay individuals explicitly commented that their belief was influenced by their personal experiences, whereas the academic psi group did not highlight a connection. A previous survey with psi researchers did reveal that personal experience was seen as persuasive for establishing the reality of psi, but less so than the cumulative experimental psi evidence (Irwin, 2014).

Overall, these findings suggest a distinction between individuals actively engaged in academic psi research and those who are not but have a strong interest and belief in psi. Although this distinction is rarely or never made in research that focuses on believers' cognition, including cognitive styles (Gray and Gallo, 2016), it is an important one for both the proponents of psi research and its skeptics. Psi researchers rightfully view their public image as one of the major hurdles facing their field (Irwin, 2014). Thus, any evidence challenging the “deficit hypothesis” as it relates to their own cognition about the legitimacy of psi phenomena should be highlighted. On the other hand, skeptics' engagement with psi research, which is increasingly finding its way into psychology and related journals (Bösch et al., 2006; Bem, 2011; Cardeña, 2018; Freedman et al., 2023), will benefit from viewing psi researchers as fellow academics who may disagree rather than individuals prioritizing belief over evidence (Reber and Alcock, 2020).

### Association between belief in psi and actively open-minded thinking

Across our entire sample encompassing diverse groups in terms of belief in psi and involvement in related research, AOT showed small-to-medium inverse correlations with psi belief and experiences. The direction of this relationship suggests that people who endorse beliefs in psi are less likely to endorse the principles of good thinking about evidence, including willingness to seek out evidence that contradicts their beliefs, to update their beliefs with new evidence, and to be comfortable with ambiguity. This association has been demonstrated previously, using heterogeneous measures of AOT and psi belief, in both undergraduate and adult samples (Pennycook et al., 2020; Rizeq et al., 2021; Newton et al., 2023). Notably, our participant selection differed not only in terms of demographics but also with the purposeful sampling at the ends of the psi belief spectrum. Relatedly, in our data, this association appears to be driven by the skeptic groups and is even stronger among them, but is virtually null within the psi groups. This suggests that the inverse relationship between actively open-minded thinking and belief in psi may not be universal, particularly among individuals with strong psi beliefs, which may be influenced by other factors.

## Limitations

Our study has limitations that are worth noting, including some pertaining to the selection of participants. The samples of academic psi and academic skeptic individuals are likely representative of their underlying populations. However, participants in the lay groups may be different from non-selected individuals from the general population who may hold belief or skepticism toward psi, as the former were recruited through venues where they actively pursued their interests and appear to be highly educated compared to the general population. Additionally, participants in the different groups were not matched on demographics, but we also presented group comparisons that took into account differences in demographics. Finally, we acknowledge limitations in the variability of some of our measures. In terms of AOT, on average, participants in all groups generally “agreed” with the principles of good thinking about evidence. In terms of psi beliefs, most scores clustered at the ends of the belief spectrum, reflecting the selection criteria for our study groups. Despite the limited ranges of these measures, we identified significant associations and differences.

The findings of this study should be interpreted in the context of the effect sizes we are able to detect with our sample. Our power analysis indicated that we can reliably detect effect sizes in the medium range or higher. When comparing the cognitive styles of psi researchers to those of skeptics, particularly academic ones, we did not find significant differences within that detectable range. It is possible that differences of a smaller magnitude exist between the groups. However, prior research has shown that differences in cognitive styles that are associated with more objective reasoning measures are typically of medium magnitude (Stanovich and West, 1997). Therefore, if small differences in cognitive styles do exist between the groups, they are unlikely to be of practical significance.

## Conclusion and future directions

Here we presented a unique comparison of cognitive styles among groups that differ in belief in psi and involvement in psi research. Our study contributes to a more nuanced understanding of the role that cognitive styles, particularly actively open-minded thinking and the need for closure, may play in the formation of psi beliefs. Additionally, it explores related differences and similarities between researchers and academics who are engaged in psi research and (1) lay believers or (2) skeptics. The cognitive styles explored here measure dispositions toward good thinking and they are markers, but not direct measures, of the ability to think critically. Future investigations could probe deeper into other aspects of cognition (including task-based) to fully examine the range of potential differences among groups, especially between academic psi researchers and academic skeptics. In addition to cognitive differences between them, other influences on psi beliefs should be explored further, as scientists, just like humans in general, have personal and sometimes strong beliefs that may impact their opinions, in addition to empirical and theoretical considerations (Coll and Taylor, 2004). Finally, given the null association found between actively open-minded thinking and psi belief at high levels of belief, future studies could investigate this relationship along the entire range of these variables. Additionally, exploring the reasons behind this differential finding could provide further insights into the development and maintenance of psi beliefs.

## Data availability statement

The raw data supporting the conclusions of this article will be made available by the authors, without undue reservation.

## Ethics statement

The studies involving humans were approved by the University of Virginia's Institutional Review Board for Social and Behavioral Sciences (protocol #3926). The studies were conducted in accordance with the local legislation and institutional requirements. The participants provided their written informed consent to participate in this study.

## Author contributions

MP: Writing – review & editing, Writing – original draft, Visualization, Validation, Software, Resources, Methodology, Investigation, Funding acquisition, Formal analysis, Data curation, Conceptualization. MW: Writing – review & editing, Writing – original draft, Visualization, Validation, Methodology. BG: Writing – review & editing, Writing – original draft, Supervision, Project administration, Investigation, Funding acquisition, Conceptualization.

## Footnotes

^1^The academic skeptic sample was recruited almost exclusively through the pool of CSI fellows. The lay skeptic sample was recruited partially through CSI affiliates or connections. Participants in the LS group are individuals who have pursued their interest in skepticism through avenues such as the Skeptical Inquirer, connections with other skeptics, or Facebook skepticism groups, but have not contributed to scientific skepticism through “distinguished” academic and/or communication efforts.

^2^Among the academic skeptic group, there were 7 respondents who endorsed some involvement in psi-related research. As an example, one respondent listed their long-term involvement with an international committee investigating paranormal claims as “pseudoscience.” Another had been involved in psi research for decades before openly stating skeptical views, questioning both the reality of the phenomena and the value and validity of the research itself.
